# Neurologic manifestations of COVID-19 and viral test in cerebrospinal fluid

**DOI:** 10.1371/journal.pone.0312621

**Published:** 2025-03-19

**Authors:** Carla de Oliveira Cardoso, Evandra Strazza Rodrigues Sandoval, Lilian Beatriz Moreira de Oliveira Chagas, Soraya Jabur Badra, Dimas Tadeu Covas, Simone Kashima Haddad, Luiz Tadeu Moraes Figueiredo

**Affiliations:** 1 Virology Research Center, Department of Internal Medicine, Medical School, University of São Paulo, Ribeirão Preto, São Paulo, Brazil; 2 Advanced Molecular Biology Laboratory, Blood Center of Ribeirão Preto, Medical School, University of São Paulo, Ribeirão Preto, São Paulo, Brazil; University of Pittsburgh Medical Center Pinnacle Health Medical Services, UNITED STATES OF AMERICA

## Abstract

**Background:**

Neurological manifestations are present in about one-third of COVID-19 cases, ranging from mild symptoms, such as anosmia, to more severe forms like demyelinating syndromes. Although direct invasion of the CNS has been demonstrated, the immune- mediated pathway is also described and more accepted. Even in cases where viral detection in CSF is absent, it should not rule out neuroinvasion. There are few prospective studies about neurological manifestations of COVID-19, especially with viral tests in CSF; as well there are still many questions about COVID-19 associated with neurological disease. Thus, we describe clinical and CSF findings of a prospective cohort of patients with nasal positive tests for SARS-CoV-2 and neurological involvement. We also discuss the pathogenic mechanisms related to these manifestations.

**Methods and Findings:**

This is a prospective cohort study; 27 patients were evaluated according to clinical presentation, the time interval between COVID-19 diagnosis and onset of neurological alterations, syndromic diagnosis, imaging and CSF findings. Real time polymerase chain reaction for SARS-CoV-2 genome was performed in all CSF samples. 2 RT-PCR in spinal cord fluid resulted positive in 9 (33.3%) cases, five of them had a positive swab nasal test concomitant to neurologic disease. Respiratory signs were described in 12 out 27 patients, five of them with viral detection in CSF. White cell counts in CSF were normal range in the majority of cases, except for 3 occurrences: two patients had elevated CSF WBC counts and viral detection in CSF (10 and 36 cells/mm3) and one also had elevated CSF WBC count but viral detection in CSF was negative (21cells/mm3). The observed neurological signs encompassed a diverse neurologic spectrum, including seizures, paresis, gait abnormalities, headaches, alteration in consciousness and memory or cognitive impairment. Both imaging and CSF alterations exhibited non-specific characteristics. Syndromic diagnoses included stroke, dementia or cognitive impairments, Guillain-Barré Syndrome, encephalitis, encephalomyelitis, acute flaccid palsy and optical neuritis.

**Conclusions:**

The patients in the present study had COVID-19 and neurologic involvement including a wide range of clinical manifestations. SARS-CoV-2 was detected in one-third of CSF samples, regardless of time interval between COVID-19 diagnosis and the onset of neurological signs. These conditions encompass various pathogenic pathways and the neuroinvasion potential of SARS-CoV-2 should be more studied.

## Introduction

In December 2019, the first case of COVID-19 (coronavirus disease-19) emerged in Wuhan, China, and it quickly reached pandemic status. Although it is known as acute respiratory disease, the involvement of other organs and systems has been well-demonstrated [1–5]. Neurological manifestations are present in about one-third of COVID-19 cases, ranging from mild symptoms, such as anosmia, to more severe forms like encephalitis and demyelinating syndromes [6]

Neurological involvement of COVID-19 affects both central nervous system (CNS) and peripheral nervous system (PNS) [7]. They can be nonspecific, such as headache, anosmia, dysgeusia, seizures and consciousness alterations, or specific, like Guillain-Barré syndrome, transverse myelitis, encephalitis, CNS thrombosis, stroke, optical neuromyelitis [8–12]. These manifestations occur even during the typical respiratory phase of COVID-19 or within a few days to two months after the respiratory disease [13]. In some cases, neurological symptoms are the initial presentation of COVID-19 [14].

The causative agent of COVID-19, Severe Acute Respiratory Syndrome Coronavirus 2 (SARS-CoV-2), attaches its Spike protein to the angiotensin-converting enzyme 2 (ACE2) receptor of the host cell for internalization. This receptor is not only abundant in lung cells but, it is also present in astrocytes, oligodendrocytes, and neurons [15]. The virus accesses the CNS through retrograde axonal transport via olfactory bulb or the adjacent cribriform plate, vagus nerve, or through proximity by interacting with ACE2 receptors on the endothelial wall of brain capillaries and nearby neural cells [16]. The hematogenous and the “Trojan horse” routes are also described [15,17].

Although direct invasion of the CNS has been demonstrated for SARS-CoV-2 and also for its predecessors (SARS-CoV-1 and MERS-CoV) [17], an immune-mediated damage pathway is also described [18]. In this model, an exacerbated immune response and inflammatory process could lead to blood-brain barrier disruption, allowing transport of inflammatory cytokines such as IL-1β, TNF-α, and IL-6, and antibodies (like anti-myelin antibodies) to CNS, causing neurological damage even after the respiratory illness has subsided [2,3,19,20].

The presence of SARS-CoV-2 in CSF has been considered direct evidence of neuroinvasion [18,21,22]. However, most publications about neurological forms of COVID-19 are case reports in which viral tests in CSF were not performed or tested negative. Perhaps the lack of this information has led to greater acceptance of the immune-mediated mechanism for neurological COVID-19 [17,18,23]. But even in cases in which viral detection in CSF is absent, it should not rule out neuroinvasion given others imaging and CSF abnormalities have been described in these cases, as well as SARS-CoV-2 genome has also been reported in both human and animal model brain tissue [17,23].

There are others pathogenic mechanisms described for the association between COVID-19 and neurologic manifestations, such as reactivation of latent virus in CNS, angiotensin-renin system dysregulation with consequent thrombotic events leading to strokes [15,24], and the hypoxemic status resultant of respiratory distress leading to neural cells damage [14,25].

There are few prospective studies about neurological manifestations of COVID-19, especially those whose diagnosis was based on viral tests in CSF; besides, there are still many questions about CSF, radiographic patterns and pathways involved in the association between neurological disease andCOVID-19. Thus, we describe here clinical, imaging and CSF findings of a prospective cohort of patients with nasal positive tests for SARS-CoV-2 and neurological involvement, admitted in a tertiary center in Ribeirão Preto, Brazil, and discuss the pathogenic mechanisms related to these manifestations.

## Methods

### Casuistic

This study is part of an ongoing cohort research (beginning in 2018), which purpose is to evaluate the viral etiology of patients with acute neurological infections admitted at Ribeirão Preto Medical School Hospital, Brazil. It was approved by local and national ethical committees (registration number: 2.790.361), and formal consent was obtained from all patients or their legal guardians. The study was funded by Fundação de Amparo à Pesquisa de São Paulo (FAPESP).

Inclusion criteria were patients with acute neurological problems attributed to COVID-19 by health care providers, who were admitted between December 1^st^, 2020 and December 31, 2022. All of them tested positive for SARS-CoV-2 in the nasal swab, CSF lumbar puncture was performed in the first week of neurological symptoms, and another viral etiology was excluded following the ongoing cohort viral test protocol that included 29 distinct viruses. Patients who did not have confirmed COVID-19 and those who did not have cerebrospinal fluid collected in referred period were excluded. Nasal swabs were taken from all patients before admission, in accordance with local regulations at the time.

Clinical data was collected from medical records, including sex, age, comorbidities, neurological signs, clinical outcomes, syndromic diagnosis, occurrence of respiratory symptoms and its time interval from neurological manifestation. Complementary exams like imaging and cytological and biochemical CSF findings were also obtained. Due to the fact these patients are part of another ongoing study, they were followed until May 2023. None of the patients had a history of neurological disease or neurological symptoms.

### Viral test in CSF

All CSF samples were tested for qualitative RNA genome of SARS-CoV-2 by real-time transcriptase reverse-polymerase chain reaction (RT-PCR) utilizing the GeneFinder^®^ COVID-19 FAST RealAmp kit (Osang HealthCare Co, Korea), approved for emergency use by Brazilian Health Agency (registration number 81914040006). The reaction was performed following the manufacturer’s instructions. RNA was extracted from 200µl of CSF samples with Mini Spin Virus DNA/RNA Extraction Kit *Biopur extraction Kit* (Mobius LifeScience^®^) according to manufacturer’s instructions.

### Analysis

The Chi-square test was used to compare percentages and means, respectively. Odds ratio with a 95% confidence interval was used to assess the association between clinical and laborator data, and the neurological syndrome presented by the patient, with the presence of SARS-CoV-2 in CSF Significance was considered at p < 0.05. Descriptive multivariate analysis was performed.

## Results

A total of 27 patients were included in the study, all with suspected neurologic manifestations of COVID-19 and positive SARS-CoV-2 RNA in nasal swab specimens. Fourteen patients were male and 13 were female; age varied between 0-82 years (median 40.85), and most of them (n = 14) were between 36-50 years old, although there was no statistical significance in this finding.

Symptoms occurred concomitantly with positive nasal test or until 60 days after that (median 14 days). RT-PCR in spinal cord fluid (CSF-RT-PCR) resulted positive in 9 (33.3%) cases, five of them had a positive swab nasal test concomitant to neurologic disease. Among negative CSF-RT-PCR patients, six presented positive nasal tests during neurologic manifestations.

Clinical findings in the 27 patients included: paresis 11(40.7%), seizures 12 (44.4%), gait abnormalities 8 (29.6%), fever 8 (29.6%), headache 7 (25.9%), consciousness alteration 7 (25.9%), memory deficits 6 (22.2%), paresthesia 6 (22.2%), palsy 3 (11.1%), speech disorder 3 (11.1%), and less commonly visual impairment, neck stiffness and vomiting (one case of each (3.7%). The arithmetic means of symptoms presented was 3.8 per patient, (variation 1-7 symptoms) with no difference between positive or negative CSF- RT-PCR. CSF-RT-PCR was positive in 4 out 11 patients with paresis, 4 out 12 patients with epilepsy, 4 out 8 patients with gait abnormalities, 4 out 8 patients with fever, 1 out 7 patients with headache, 2 out 4 patients with paresthesia, 1 out 7 patients with consciousness alteration, 2 out 6 patients with memory or cognitive deficits and 2 out 3 with speech disorders. Those patients with a positive CSF-RT-PCR did not experience palsy, visual impairment, vomiting or delirium. Therefore, viral detection in CSF was not related to the neurological manifestations associated with COVID-19, as shown in Table 1. Respiratory complaints were expressed in 12 out 27 patients, five with positive CSF-RT-PCR. Comorbidities were significantly higher in the positive CSF-RT-PCR group (p < 0.006459), including hypertension, obesity and type 2 diabetes. Immunosuppressive condition was found in 2 cases: one positive and another negative CSF-RT-PCR.

**Table 1:** Clinical characterization of patients with neurologic manifestations of COVID- 19 according to CSF-RT-PCR results.

Characterization	Positive CSF-RT-PCR	Negative CSF-RT-PCR
Sex		
Male	6 (66.6%)	8 (44.4%)
Female	3 (33.3%)	10 (66.6%)
Age		
0-18y	2 (22.2%)	4 (22.2%)
19-35y	2 (22.2%)	2 (11.1%)
36-60y	4 (44.4%)	10 (55.5%)
> 60y	1 (11.1%)	2 (11.1%)
Total	9 (100%)	18 (100%)
Clinical signs		
Paresis	4 (44.4%)	7 (38.9%)
Seizures	4 (44.4%)	8 (44.4%)
Gait abnormalities	4 (44.4%)	4 (22.2%)
Fever	4 (44.4%)	4 (22.2%)
Headache	1 (11.1%)	6 (33.3%)
Paresthesia	2 (22.2%)	4 (22.2%)
Consciousness alteration	1 (11.1%)	6 (33.3%)
Memory or cognitive deficits	2 (22.2%)	4 (22.2%)
Palsy	none	3 (16.6%)
Visual impairment	none	1(5.5%)
Vomiting	none	1(5.5%)
Speech disorder	2 (22.2%)	1(5.5%)
Neck stiffness	1(11.1%)	none
Coma	1 (11.1%)	2 (11.1%)
Delirium	none	3 (16.6%)
Respiratory symptoms	5 (66.6%)	7 (38.9%)
Immunosuppressive condition	1 (11.1%)	1(5.5%)
Comorbidities^*^	8 (88.9%)	6 (33.3%)

* p < 0.0006459 (CI 95%).

Considering imaging findings, 14 patients had no abnormalities in nuclear magnetic resonance or tomography; eleven (40.7%) presented abnormalities like hypodensities, cerebral atrophy, foci of demyelination, inflammatory and hemorrhagic process; two cases had no imaging. Only 2 patients had imaging abnormalities that could be due to a previous undiagnosed condition, both with CSF-RT-PCR negative. None of them had previous imaging records nor neurological disease previously diagnosed. The neurological condition of all these patients was attributed to COVID-19 after excluding other possible causes. White cell counts in CSF were normal (reference: <  5 cells/mm^3^ after 1 month age) in all cases except for 3 adults: two of them were positive in CSF-RT-PCR (10 and 36 cells/mm^3^) and one was negative (21 cells/mm^3^). Glucose levels (reference: > 50md/dl or < 2/3 blood sugar result) were normal in all samples and protein levels were slightly elevated (reference: <  44 md/dl) in 12 samples: four patients with positive CSF-RT-PCR (69-85 mg/dl) and 8 with negative CSF-RT-PCR (49-469mg/dl). (Table 2).

**Table 2:** Imaging and CSF findings in patients with neurologic manifestation of COVID-19, according to SARS-CoV-2 CSF-RT-PCR result.

Findings	Positive CSF PCR	Negative CSF PCR
Imaging		
Normal	2 (22.2%)	12(66.6%)
Alterated	6 (66.6%)	5 (27.8%)
No record	1(11.1%)	1(5.6%)
CSF white cell count		
0-5cel/mm3	6 (66.6%)	17 (94.4%)
6-10 cel/mm3	1(11.1%)	none
11-50 cel/mm3	1(11.1%)	1(5.6%)
Not counted	1(11.1%)	none
CSF protein		
Normal	5 (55.6%)	10 (66.6%)
Elevated	4 (44.4%)	8 (44.4%)
Total	9 (100%)	18 (100%)

Nineteen patients had poor clinical outcomes: two died and 17 evolved with sequelae or chronic conditions (5 positive and 12 negative CSF-RT-PCR testing). As shown in Table 3, syndromic diagnosis included stroke, epileptic manifestations, meningoencephalitis, encephalomyelitis, nonspecific dementia, Guillain-Barré Syndrome, optical neuritis, acute flaccid palsy, polyneuropathy, encephalitis and headache.

**Table 3:** Syndromic diagnosis of patients with neurological forms of COVID-19 according to CSF-RT-PCR result.

Diagnosis	Positive CSF-RT-PCR	Negative CSF-RT-PCR
	**Positive**	**Negative**
Stroke	2 (22.2%)	1 (5.5%)
Nonspecific dementia	2 (22.2%)	2(11.1%)
Seizures/epilepsy	2 (22.2%)	6 (33.3%)
Guillain-Barré Syndrome	1(11.1%)	1 (5.5%)
Meningoencephalitis	1(11.1%)	none
Encephalomyelitis	1(11.1%)	none
Acute flaccid palsy	none	2 (11.1%)
Polyneuropathy	none	1 (5.5%)
Optical neuritis	none	1 (5.5%)
Headache	none	1 (5.5%)
Encephalitis	none	2(11.1%)
Meningitic signs	none	1 (5.5%)
Total	9 (100%)	18 (100%)

## Discussion

In this prospective cohort study, all patients presenting distinct neurologic involvement associated with COVID-19 had their CSF samples tested for the SARS-CoV-2 genome. It drew our attention that most publications on neurologic manifestations associated with COVID-19 are case reports or review articles in which viral tests in CSF are not described. Our study brings together CSF-RT-PCR, clinical, imaging and CSF features. Twenty-seven patients participated in this study, one of the most significant samples reported [22,26,27].

Detection rates of SARS-CoV-2 in CSF samples are low, ranging from 4.76 to 9% of samples tested [21,27,28]. Our positivity rate to SARS-CoV-2 in CSF reached 33.3%, much higher than has been reported. This result is probably related to the viral load in CSF, the time interval between the appearance of the first symptoms and lumbar puncture, or the high sensitivity of the PCR kit we utilized (limit of detection of 2 copies/µl for gene N and five copies/µl for genes RdRP and E). The lack of standardized protocols may also be the cause of discrepant results [15,25].

There is a wide range of neurologic manifestations associated with COVID-19, occurring from isolated signs to well-known neurological syndromes that evolve from distinct pathogenic mechanisms [4,8,10,11,29,30]. We could not correlate such manifestations with the time interval between respiratory symptoms (when they existed) or viral detection in nasal swabs, as similar manifestations could be observed either concomitant with COVID-19 or after convalescence of the disease. Indeed, respiratory symptoms do not seem to be essential to neurological involvement, since some patients did not have any respiratory complaints, and the nasal swab was the unique evidence of COVID-19 [31]. Thus, it is probable that hypoxia, as a result of respiratory failure, would damage neural cells and lead to neurological disease, which is not a pathogenic pathway applicable to all cases [14,32].

Neurologic manifestations are described in patients with prolonged disease or late-onset sequelae of COVID-19 and they are called “Long Covid” [31]. These manifestations include brainstorm, fatigue, anosmia, dysgeusia, cognitive or memory loss, psychiatric disorders, motor autonomic or sensitive impairment, sleep disorders, Guillain- Barré Syndrome; it occurs in about one-third of COVID-19 survivors [33]. The same mechanisms involved in neurologic complications of COVID-19 are engaged in Long Covid [25,31,33]. Although neural direct invasion has been considered in the pathogenesis of Long Covid, viral persistence in CSF has not been documented [25,33]. Also, our study does not have sufficient data to confirm or deny this hypothesis.

Some authors describe that the pathogenesis of neurological involvement of COVID-19 is related to the time interval between the disease and the moment which the neurological symptoms arise: if neurological and respiratory symptoms are simultaneous, the neural invasion of SARS-CoV-2 is the mechanism more plausible, but if neurological signs arise latterly, the immune-mediated mechanism could be the causative [27,28,34]. Our data does not support this theory about the pathogenesis of COVID-19 affecting CNS, because the viral genome could be detected in CSF samples either concomitant or after COVID-19.

Detection of SARS-CoV-2 in CSF has been firmly associated with evidence of neuroinvasion [13,22]. As the detection rates in CSF described elsewhere are low, some researchers have discouraged routine tests and considered that the pathogenesis of COVID-19 and neurologic involvement could be immune-mediated. Our 33% detection rate of SARS-CoV-2 in CSF samples suggests that these tests are valid and more studies are necessary to better understand the timing and viral load of SAR-CoV-2 in CSF.

Many pathogenic ways are described associating COVID-19 and neurological disease (neuroinvasion, cytokine storm, immune-mediated mechanism, thrombotic events, cross-reaction or production of autoantibodies, effects of hypoxia status) and probably one or more of these mechanisms might be present in each case [16,25,35–37]. Until now, none of these explanations could be applied to all situations. The pathogenic pathway might be more related to the final syndromic diagnosis presented for each patient, like the production of autoantibodies observed in cases of demyelinating syndromes with anti-myelin antibodies detection, or the thrombotic events in patients with stroke or central venous thrombosis in brain.

Interestingly, it was not possible to correlate any imaging alteration to neurologic manifestations in COVID-19, neither biochemical nor cytologic pattern in CSF [24,38–40]. Although more neurological findings were observed in positive CSF-RT-PCR cases, it was not significant.

This study has some limitations, like the small sample size, localization in one single center, and the presence of a low number of immunosuppressed patients. Besides, inflammatory/ immune markers, like oligoclonal bands, immunoglobulin G index in CSF, auto-antibodies like anti-myelin and aquaporin 4, and interleukin-6 were not performed in all patients. The neurological condition of all these patients was attributed to COVID-19. Some patients presented neurological signs a long time after testing positive for SARS-CoV-2 (up to 60 days). Although it may be questioned whether COVID-19 was really the cause of the neurological condition, other potential causes have been ruled out previously.

However, it is a cohort study on neurologic manifestations of COVID-19 that includes the detection of SARS-CoV-2 in CSF associated with clinical, imaging, and CSF parameters. Our results suggest that SARS-CoV-2 indeed invades CNS and more studies are necessary to better understand this mechanism.

## Conclusion

In conclusion, the patients in the present study had COVID-19 and neurologic involvement including a wide range of clinical manifestations. SARS-CoV-2 was detected in one-third of CSF samples, regardless of the time interval between COVID-19 and the onset of neurological signs. The neuroinvasion potential of SARS-CoV-2 should be more studied.

## References

[1] Borra R, Patel NT, Shaikh R, Parmar MS, Borra S. A case of transverse myelitis secondary to COVID-19 infection. Cureus. 2022;14(12):e32297. doi: 10.7759/cureus.32297 36628040 PMC9822530

[2] Noon A, Malhi J, Wong C. Atypical guillain-barré syndrome presenting after COVID-19 infection. Cureus. 2022;14(9):e29521. 36312645 10.7759/cureus.29521 PMC9589521

[3] Doukas SG, Santos AP, Mir W, Daud S, Zivin-Tutela TH. A rare case of myelin oligodendrocyte glycoprotein antibody-associated transverse myelitis in a 40-year-old patient with COVID-19. Cureus. 2022;14(4):e23877. doi: 10.7759/cureus.23877 35530898 PMC9074907

[4] Siegler J, Dasgupta S, Abdalkader M, Penckofer M, Yaghi S, Nguyen T. Cerebrovascular disease in COVID-19. Viruses. 2023;15(7):1598. 37515284 10.3390/v15071598 PMC10385090

[5] Jansen van Vuren E, Steyn S, Brink C, Möller M, Viljoen F, Harvey B. The neuropsychiatric manifestations of COVID-19: interactions with psychiatric illness and pharmacological treatment. Biomed Pharmacother. 2021;135:111200. 33421734 10.1016/j.biopha.2020.111200 PMC7834135

[6] Mao L, Jin H, Wang M, Hu Y, Chen S, He Q, et al. Neurologic manifestations of hospitalized patients with coronavirus disease 2019 in Wuhan, China. JAMA Neurol. 2020;77(6):683–90. doi: 10.1001/jamaneurol.2020.1127 32275288 PMC7149362

[7] Börü ÜT, Bölük C, Toksoy CK, Demirbaş H. Acute cerebellitis, transverse myelitis and polyradiculoneuritis related to post-COVID-19 infection. J Spinal Cord Med. 2022;45(5):765–8. doi: 10.1080/10790268.2021.1969502 36175361 PMC9542541

[8] Huo L, Xu K-L, Wang H. Clinical features of SARS-CoV-2-associated encephalitis and meningitis amid COVID-19 pandemic. World J Clin Cases. 2021;9(5):1058–78. doi: 10.12998/wjcc.v9.i5.1058 33644169 PMC7896657

[9] Mondal R, Deb S, Shome G, Ganguly U, Lahiri D, Benito-León J. COVID-19 and emerging spinal cord complications: A systematic review. Mult Scler Relat Disord. 2021;51:102917. doi: 10.1016/j.msard.2021.102917 33845350 PMC7981271

[10] Heuser K, Berger TC, Henning O, Svalheim S, Sibeko JM, Nakken KO, et al. COVID-19 and epilepsy. Tidsskr Den Nor Legeforening [Internet]. 28 de junho de 2021 [citado 24 de agosto de 2023]; Disponível em: https://tidsskriftet.no/en/2021/06/debatt/covid-19-and-epilepsy

[11] Vohora D, Jain S, Tripathi M, Potschka H. COVID-19 and seizures: Is there a link?. Epilepsia. 2020;61(9):1840–53. doi: 10.1111/epi.16656 32944929 PMC7537056

[12] Sancho-Saldaña A, Lambea-Gil Á, Liesa JLC, Caballo MRB, Garay MH, Celada DR, et al. Guillain-Barré syndrome associated with leptomeningeal enhancement following SARS-CoV-2 infection. Clin Med (Lond). 2020;20(4):e93–4. doi: 10.7861/clinmed.2020-0213 32518103 PMC7385766

[13] Cautilli F, Feleppa M, Valeriani M, Papetti L, Monte G, Midulla F, et al. Case report: A case of acute disseminated encephalomyelitis after SARS-CoV-2 infection in pediatric patients. Front Neurol. 2023;14:1099458. doi: 10.3389/fneur.2023.1099458 36908623 PMC9992531

[14] Palahuta HV, Fartushna OY, Yevtushenko SK, Hnepa YY. Acute transverse myelitis as a neurological complication of COVID-19: A case report. Wiadomosci Lek. 2021;74(4):1045–9. 34156028

[15] Divani A, Andalib S, Biller J, Di Napoli M, Moghimi N, Rubinos C. Central nervous system manifestations associated with COVID-19. Current Neurology and Neuroscience Reports. 2020;20(12):60. 33128130 10.1007/s11910-020-01079-7 PMC7599061

[16] Galea M, Agius M, Vassallo N. Neurological manifestations and pathogenic mechanisms of COVID-19. Neurol Res. 2022;44(7):571–82. doi: 10.1080/01616412.2021.2024732 34986754

[17] Ismail II, Salama S. Association of CNS demyelination and COVID-19 infection: an updated systematic review. J Neurol. 2022;269(2):541–76. doi: 10.1007/s00415-021-10752-x 34386902 PMC8359762

[18] Tuma RL, Guedes BF, Carra R, Iepsen B, Rodrigues J, Camelo-Filho AE, et al. Clinical, cerebrospinal fluid, and neuroimaging findings in COVID-19 encephalopathy: a case series. Neurol Sci. 2021;42(2):479–89. doi: 10.1007/s10072-020-04946-w 33409828 PMC7787933

[19] Letchuman V, Wemhoff KM, Gandhoke GS. Acute inflammatory demyelinating polyneuropathy with bowel and bladder incontinence following COVID-19 infection. Cureus. 2021;13(9):e17896. doi: 10.7759/cureus.17896 34660094 PMC8505905

[20] Alrubaye R, Bondugula V, Baleguli V, Chofor R. A possible Guillain-Barré syndrome/transverse myelitis overlap syndrome after recent COVID-19. BMJ Case Rep. 2022;15(2):e246967. doi: 10.1136/bcr-2021-246967 35140089 PMC8830199

[21] Zamani R, Pouremamali R, Rezaei N. Central neuroinflammation in Covid-19: a systematic review of 182 cases with encephalitis, acute disseminated encephalomyelitis, and necrotizing encephalopathies. Rev Neurosci. 2022;33(4):397–412 34536341 10.1515/revneuro-2021-0082

[22] Lewis A, Frontera J, Placantonakis DG, Lighter J, Galetta S, Balcer L, et al. Cerebrospinal fluid in COVID-19: A systematic review of the literature. J Neurol Sci. 2021;421:117316. doi: 10.1016/j.jns.2021.117316 33561753 PMC7833669

[23] Erickson MA, Rhea EM, Knopp RC, Banks WA. Interactions of SARS-CoV-2 with the Blood-Brain Barrier. Int J Mol Sci. 2021;22(5):2681. doi: 10.3390/ijms22052681 33800954 PMC7961671

[24] Yeahia R, Schefflein J, Chiarolanzio P, Rozenstein A, Gomes W, Ali S, et al. Brain MRI findings in COVID-19 patients with PRES: A systematic review. Clin Imaging. 2022;81:107–13. doi: 10.1016/j.clinimag.2021.10.003 34700172 PMC8519663

[25] Monje M, Iwasaki A. The neurobiology of long COVID. Neuron. 2022;110(21):3484–96. doi: 10.1016/j.neuron.2022.10.006 36288726 PMC9537254

[26] Feizi P, Sharma K, Pasham SR, Nirwan L, Joseph J, Jaiswal S, et al. Central nervous system (CNS) inflammatory demyelinating diseases (IDDs) associated with COVID-19: A case series and review. J Neuroimmunol. 2022;371:577939. doi: 10.1016/j.jneuroim.2022.577939 35939945 PMC9343076

[27] Destras G, Bal A, Escuret V, Morfin F, Lina B, Josset L, et al. Systematic SARS-CoV-2 screening in cerebrospinal fluid during the COVID-19 pandemic. Lancet Microbe. 2020;1(4):e149. doi: 10.1016/S2666-5247(20)30066-5 32835345 PMC7289579

[28] Jarius S, Pache F, Körtvelyessy P, Jelčić I, Stettner M, Franciotta D, et al. Cerebrospinal fluid findings in COVID-19: a multicenter study of 150 lumbar punctures in 127 patients. J Neuroinflammation. 2022;19(1):19. doi: 10.1186/s12974-021-02339-0 35057809 PMC8771621

[29] Miners S, Kehoe P, Love S. Cognitive impact of COVID-19: looking beyond the short term. Alzheimer’s Res Ther. 2020;12:170. doi: 10.1186/s13195-020-00732-5 33380345 PMC7772800

[30] Andalib S, Biller J, Di Napoli M, Moghimi N, McCullough L, Rubinos C. Peripheral nervous system manifestations associated with COVID-19. Curr Neurol Neurosci Rep. 2021;21(3):9. 33586020 10.1007/s11910-021-01102-5 PMC7882462

[31] Stefanou M-I, Palaiodimou L, Bakola E, Smyrnis N, Papadopoulou M, Paraskevas GP, et al. Neurological manifestations of long-COVID syndrome: a narrative review. Ther Adv Chronic Dis. 2022;13:20406223221076890. doi: 10.1177/20406223221076890 35198136 PMC8859684

[32] Hingorani KS, Bhadola S, Cervantes-Arslanian AM. COVID-19 and the brain. Trends Cardiovasc Med. 2022;32(6):323–30. doi: 10.1016/j.tcm.2022.04.004 35461991 PMC9022395

[33] Leng A, Shah M, Ahmad SA, Premraj L, Wildi K, Li Bassi G, et al. Pathogenesis underlying neurological manifestations of long COVID syndrome and potential therapeutics. Cells. 2023;12(5):816. doi: 10.3390/cells12050816 36899952 PMC10001044

[34] Krasemann S, Haferkamp U, Pfefferle S, Woo MS, Heinrich F, Schweizer M, et al. The blood-brain barrier is dysregulated in COVID-19 and serves as a CNS entry route for SARS-CoV-2. Stem Cell Rep. 2022;17(2):307–20. doi: 10.1016/j.stemcr.2021.12.011 35063125 PMC8772030

[35] Vanderheiden A, Klein R. Neuroinflammation and COVID-19. Curr Opin Neurobio. 2022;76:102608. 10.1016/j.conb.2022.102608 PMC9239981 35863101

[36] Skripuletz T, Möhn N, Franke C, Prüß H. Neuroimmunologie von COVID‑19. Nervenarzt. 2021;92(6):521–30. 33651117 10.1007/s00115-021-01077-1 PMC7923405

[37] Gombolay G, Anderson M, Xiang Y, Bai S, Rostad C, Tyor W. Neurological complications in children hospitalized with seizures and respiratory infections: A comparison between SARS-CoV-2 and other respiratory infections. Pediatric Neurology. 2022;135:52–5. 35995010 10.1016/j.pediatrneurol.2022.07.010 PMC9338832

[38] Petersson I, Hansen BM, Svenningsson A, Lundstrom A. Cerebral microvascular injuries in severe COVID-19 infection: progression of white matter hyperintensities post-infection. BMJ Case Rep. 2022;15(9):e249156. doi: 10.1136/bcr-2022-249156 36100286 PMC9472107

[39] Barman A, Sahoo J, Viswanath A, Roy SS, Swarnakar R, Bhattacharjee S. clinical features, laboratory, and radiological findings of patients with acute inflammatory myelopathy after COVID-19 infection: a narrative review. Am J Phys Med Rehabil. 2021;100(10):919–39. doi: 10.1097/PHM.0000000000001857 34347629 PMC8436817

[40] Sriwastava S, Tandon M, Podury S, Prasad A, Wen S, Guthrie G, et al. COVID-19 and neuroinflammation: a literature review of relevant neuroimaging and CSF markers in central nervous system inflammatory disorders from SARS-COV2. J Neurol. 2021;268(12):4448–78. doi: 10.1007/s00415-021-10611-9 34009454 PMC8131883

