# Supplementary material for: Neurologic manifestations of COVID-19 and viral test in cerebrospinal fluid
Source: PLoS One. 2025 Mar 19;20(3):e0312621. doi: 10.1371/journal.pone.0312621 (PMC11922214; doi:10.1371/journal.pone.0312621)
Supplement: Attachment — Submitted filename: RESPONSE TO REVIEWERS.docx [file pone.0312621.s001.docx]

Ribeirão Preto, February 15, 2024

Staff Editors

Plos One

Dear Editors:

We have submitted our manuscript **Neurologic manifestations of COVID-19 and viral test in cerebrospinal fluid,** where we studied the neurological involvement in COVID-19 and the presence of SARS-CoV-2 in cerebrospinal fluid, besides its association with clinical features, syndromic diagnosis, radiographic findings, biochemical and cell parameters in CSF.

Here, we response to Journal requirements:

1- We have adjusted file names to meet Plos One’s style requirement;
2- A) The cohort study was supported by Fundação de Amparo à Pesquisa de São Paulo, which maintains almost all researches from Virology Research Center. The Foundation is not involved in the design of the project, the collection or analysis of data, and is not involved in the publication of the final work. Neither I nor the other authors have been paid by this foundation for their work;

B) “the funders had no role in study design, data collection and analysis, decision to publish, or preparation of the manuscript”;

C, D) “the authors received no specific funding for this work”;

3- We confirm that all data are within the manuscript, and also confirm the reviewers;
4- The reference list was updated.

Here, we answer the Reviewer’s considerations:

Reviewer 1

1- The exclusion criteria were patients who have not been diagnosed with COVID-19 and patients who refused to participate in the study. Patients were included in the study group at the time we received CSF samples, so patients whose CSF was not collected were excluded;
2- During the pandemia, the nasal test was carried out for all patients admitted to the hospital according to internal procedures. This information was included in the text;
3- The reference parameters for glucose, protein and cell in the CSF were included in the text;
4- The table and line 155 have been modified. All three patients with CSF leukocytosis were adults and these data are included in the text as a reference value;
5- The errors have been identified and we hope that they will all be corrected.

Reviewer 2

1- Abstract: lines 33, 39, 45, 46 and 47 were fixed.
2- Introduction

- Lines 74, 75, 76 and 115 were fixed;

- Reference 6 has been fixed, sorry for the mistake.

3- Method

- Given the cohort criteria, patients from this work were followed up until May 2023. This information is presented in the section;

- Funding: information was included in the text; this work was funded by the Fundação de Amparo à Pesquisa de São Paulo.

4- Analysis: Descriptive multivariate analysis was performed and information was added in the text.

5- Results

- Fixed lines 163, 164, 165, 166, 167, 168, 169, 170, 176, 177, 178 and 179;

- Patients showed an average of 3.8 symptoms with no difference between groups. Further details are given in the text;

- Two imaging abnormalities may be the result of previously undiagnosed injury, both patients were negative in CSF-RT-PCR. The information is described in the text.

6- Discussion

- Lines 202, 211, 212, 213, 216, 217, 218, 219, 220, 221, 226 and 234 were modified;
- Gramatical mistakes were fixed.

Reviewer 3

- None patient had previous neurologic condition diagnosed, and none of the neurological manifestations associated with COVID-19 were considered a recrudescence by the assistant staff. This information was added in the text.

We sincerely thank all the reviewer’s contributions and we expected all the mistakes of this work were solved.

I hope that my clarifications have resolved any doubts that may exist and I am at your disposal for further information.

Please let us know if you need further information.

Yours sincerely,

Carla de Oliveira Cardoso

Virology Research Center,

Department of Internal Medicine

Ribeirão Preto Medical School

University of São Paulo,

Ribeirão Preto, São Paulo, Brazil.

Phone: 55 34 99103 9775

carla_cardoso@hotmail.com
