# Supplementary material for: Neurologic manifestations of COVID-19 and viral test in cerebrospinal fluid
Source: PLoS One. 2025 Mar 19;20(3):e0312621. doi: 10.1371/journal.pone.0312621 (PMC11922214; doi:10.1371/journal.pone.0312621)
Supplement: Attachment — Submitted filename: RESPONSE TO REVIEWERS 14 de junho de 2024.docx [file pone.0312621.s002.docx]

Ribeirão Preto, June 14, 2024

Staff Editors

Plos One

Dear Editors:

We have submitted our manuscript **Neurologic manifestations of COVID-19 and viral test in cerebrospinal fluid,** where we studied the neurological involvement in COVID-19 and the presence of SARS-CoV-2 in cerebrospinal fluid, besides its association with clinical features, syndromic diagnosis, radiographic findings, biochemical and cell parameters in CSF.

Here, we answer the Reviewer’s considerations:

1- Introduction

- Lines 185 (p-value) was fixed;

2- Results

- Information about imaging findings was discussed in the text below table 2: none of patients had a history of previous neurologic disease and we did not have imaging exams before COVID-19. Two imaging abnormalities may be the result of previously undiagnosed injury, both patients were negative in CSF-RT-PCR.

3- Discussion

- Although we think that our purpose was not to discuss aspects of Long Covid, we accepted the reviewer’s suggestion and we briefly discussed this topic. We do not have sufficient data to support viral presence in CSF in cases of Long Covid, but this mechanism is questioned in some publications.

We sincerely thank all the reviewer’s contributions and we expected all the mistakes of this work were solved.

I hope that my clarifications have resolved any doubts that may exist and I am at your disposal for further information.

Please let us know if you need further information.

Yours sincerely,

Carla de Oliveira Cardoso

Virology Research Center,

Department of Internal Medicine

Ribeirão Preto Medical School

University of São Paulo,

Ribeirão Preto, São Paulo, Brazil.

Phone: 55 34 99103 9775

carla_cardoso@hotmail.com
