# Supplementary material for: Neurologic manifestations of COVID-19 and viral test in cerebrospinal fluid
Source: PLoS One. 2025 Mar 19;20(3):e0312621. doi: 10.1371/journal.pone.0312621 (PMC11922214; doi:10.1371/journal.pone.0312621)
Supplement: Attachment — Submitted filename: RESPONSE TO REVIEWERS 12 de setembro de 2024.docx [file pone.0312621.s003.docx]

Ribeirão Preto, September 12, 2024

Staff Editors

Plos One

Dear Editors:

We have submitted our manuscript **Neurologic manifestations of COVID-19 and viral test in cerebrospinal fluid,** where we studied the neurological involvement in COVID-19 and the presence of SARS-CoV-2 in cerebrospinal fluid, besides its association with clinical features, syndromic diagnosis, radiographic findings, biochemical and cell parameters in CSF.

Here, we answer the Reviewer’s considerations:

- Lines 133-135 were fixed;

- The patient who presented neurologic signs 60days after testing positive for SARS-CoV-2 is a nurse around 40 years old. These symptoms included memory loss, sadness, and confusion while performing her work duties. It's possible that these symptoms began earlier, but the diagnosis was only made when her colleagues noticed her struggles. Still, we emphasized in the text further in both the results and discussion sections of the study that all cases were attributed to COVID-19 after ruling out other diseases.

We sincerely thank all the reviewer’s contributions and we expected all the mistakes of this work were solved.

I hope that my clarifications have resolved any doubts that may exist and I am at your disposal for further information.

Please let us know if you need further information.

Yours sincerely,

Carla de Oliveira Cardoso

Virology Research Center,

Department of Internal Medicine

Ribeirão Preto Medical School

University of São Paulo,

Ribeirão Preto, São Paulo, Brazil.

Phone: 55 34 99103 9775

carla_cardoso@hotmail.com
